# CpG island status as an epigenetic alteration for NIS promoter in thyroid neoplasms; a cross-sectional study with a systematic review

**DOI:** 10.1186/s12935-022-02720-w

**Published:** 2022-10-11

**Authors:** Maryam Zarkesh, Noman Arab, Raziyeh Abooshahab, Shabnam Heydarzadeh, Sara Sheikholeslami, Zahra Nozhat, Marziyeh Salehi Jahromi, Seyed Ahmad Fanaei, Mehdi Hedayati

**Affiliations:** 1 Cellular and Molecular Endocrine Research Center, Research Institute for Endocrine Sciences, Shahid Beheshti University of Medical Sciences, Tehran, Iran; 2 Department of Clinical Biochemistry, School of Medicine, Shahid Beheshti University of Medical Sciences, Tehran, Iran; 3 Curtin Medical School, Curtin University, Bentley, 6102 Australia; 4 Institute of Smart Biomedical Materials, School of Materials Science and Engineering, Zhejiang Sci-Tech University, Hangzhou, 310018 China; 5 Department of Physiology and Pharmacology, Center for Diabetes and Endocrine Research, College of Medicine, University of Toledo, Toledo, OH USA; 6 Erfan Hospital, Tehran, Iran; 7 Cellular and Molecular Endocrine Research Center, Research Institute for Endocrine Sciences, Shahid Beheshti University of Medical Sciences, PO Box: 19395-4763, Tehran, Iran

**Keywords:** Papillary thyroid cancer, Follicular thyroid cancer, Multinodular goiter, DNA methylation, CpG islands, Epigenetics, Sodium/iodide symporter

## Abstract

**Background:**

Gene silence via methylation of the CpG islands is cancer's most common epigenetic modification. Given the highly significant role of *NIS* in thyroid cancer (TC) differentiation, this cross-sectional study aimed to investigate the DNA methylation pattern in seven CpG islands (CpG1-7 including +846, +918, +929, +947, +953, +955, and +963, respectively) of the NIS promoter in patients diagnosed with papillary (PTC), follicular (FTC), and multinodular goiter (MNG). Additionally, a systematic review of the literature was conducted to compare our results with studies concerning methylation of the NIS gene promoter.

**Methods:**

Thyroid specimens from 64 patients met the eligibility criteria, consisting of 28 PTC, 9 FTC, and 27 benign MNG cases. The mRNA of *NIS* was tested by qRT-PCR. The bisulfite sequencing PCR (BSP) technique was performed to evaluate the promoter methylation pattern of the *NIS* gene. Sequencing results were received in chromatograph, FASTA, SEQ, and pdf formats and were analyzed using Chromas. The methylation percentage at each position and for each sample was calculated by mC/(mC+C) formula for all examined CpGs; following that, the methylation percentage was also calculated at each CpG site. Besides, a literature search was conducted without restricting publication dates. Nine studies met the eligibility criteria after removing duplicates, unrelated articles, and reviews.

**Results:**

*NIS* mRNA levels decreased in tumoral tissues of PTC (P = 0.04) and FTC (P = 0.03) patients compared to their matched non-tumoral ones. The methylation of *NIS* promoter was not common in PTC samples, but it was frequent in FTC (P < 0.05). Significant differences were observed in the methylation levels in the 4th(+ 947), 6th(+ 955), and 7th(+ 963) CpGs sites in the forward strand of *NIS* promoter between FTC and MNG tissues (76.34 ± 3.12 *vs* 40.43 ± 8.42, P = 0.004, 69.63 ± 3.03 *vs* 23.29 ± 6.84, P = 0.001 and 50.33 ± 5.65 *vs* 24 ± 6.89, P = 0.030, respectively). There was no significant correlation between the expression and methylation status of *NIS* in PTC and FTC tissues.

**Conclusion:**

Perturbation in *NIS* promoter’s methylation individually may have a potential utility in differentiating MNG and FTC tissues. The absence of a distinct methylation pattern implies the importance of other epigenetic processes, which may alter the production of *NIS* mRNA. In addition, according to the reversibility of DNA methylation, it is anticipated that the design of particular targeted demethylation medicines will lead to a novel cancer therapeutic strategy.

## Introduction

Thyroid cancers are the most common malignancies and represent approximately 95% of all endocrine tumors. The global incidence of thyroid cancer was estimated to be 586,000 cases in 2020, and nearly 43,646 patients were presumed to die from thyroid cancer [1]. Thyroid cancer comprises several subtypes with remarkably different biological characteristics. Among these subtypes, papillary and follicular thyroid cancers (PTC and FTC) are referred to as well-differentiated thyroid cancers (WDTCs), accounting for more than 90% of all thyroid malignancies [2]. WDTCs typically are well managed and represent a favorable prognosis; however, sometimes, patients progress toward poorly differentiated and more aggressive subtypes [3].

Nowadays, environmental factors, alongside genetic alterations, play a pivotal role in developing thyroid cancers [4, 5]. Ecological factors in regulating gene expression are known as epigenetic alterations [6–8]. Aberrant DNA methylation has been introduced as a major epigenetic event in tumorigenesis [9, 10]. Gene inactivation through hypermethylation of CpG islands located in promoter regions was described in many cancers, especially thyroid cancer, which correlated with progression and tumorigenesis [11].

Solute carrier family 5-member 5 (SLC5A5, also called *NIS*) gene is localized on chromosome 19 (19p13.11) and encodes an 80–90 kDa transmembrane glycoprotein that actively transports iodide from the bloodstream into the thyroid follicular cells [12, 13]. Indeed, thyroid-stimulating hormone (TSH) can regulate NIS protein activity. In this process, a Na+/K+-ATPase pump generates a sodium gradient, transporting two Na+ to an I− as the first step in the thyroid hormones biosynthesis [14]. The role of *NIS* in thyroid cancer diagnosis and treatment is important since *NIS* primarily mediates radioactive iodine (RAI) accumulation. Accordingly, the reduced *NIS* mRNA levels and/or impairment in *NIS* plasma membrane trafficking are well-demonstrated factors showing poor prognosis in thyroid cancer [15]. However, the association between *NIS* and thyroid cancer is complex and poorly understood [13].

Traditionally, it has been argued that CpG islands exist within the promoter of the *NIS* gene. In this regard, several studies have demonstrated that this island is a methylation target, which could have a vital role in DNA methylation, causing the reduction of *NIS* mRNA expression in thyroid carcinomas [12, 16–20].

In molecular cancer medicine development history, epigenetics has been thought of as a critical factor. In essence, epigenetic alterations depend on different factors, including race, environmental changes, lifestyle, nutrition, and physical activity. As above-mentioned, there are several studies regarding *NIS* promoter’s methylation in thyroid cancers. However, no previous study has investigated changes in those factors in an Iranian population as epigenetic modifications are strongly linked with race. Therefore, according to the mentioned challenges, this study seeks to investigate the methylation degree of a large region of *NIS* promoter in a set of multinodular goiters (MNG) samples (as the benign samples), malignant thyroid tumors including PTC and FTC, and their surrounding non-tumor tissue samples to suggest a methylation region as a discriminatory biomarker for early diagnosis of thyroid nodules. In addition, a systematic review of the literature was completed to bring together studies regarding methylation of *NIS* gene promoter, to reach a general conclusion about the role of *NIS* promoter methylation in tumorigeneses, and to discuss with our results.

## Methods and materials

### Tissue samples

Patients who underwent surgical resection of thyroid tumor at Erfan and Atiyeh hospitals in Tehran, Iran, were selected from 2015 to 2016. The PTC, FTC, and MNG subjects were confirmed based on pathological evidence and clinical outcomes. Histological assessment was performed using formalin-fixed, paraffin-embedded tissue specimens serially sectioned into 4–5 μm thick slices. The cryosections were examined by an expert pathologist after hematoxylin and eosin (H&E) staining to verify normal and tumoral tissues. Overall, 64 patients met the eligibility criteria, consisting of 28 PTCs, 9 FTCs cases (tumoral tissues and the matched non-tumoral tissues from the same patients), and 27 benign cases (non-tumoral tissues from patients with MNG). Tissue samples were collected in RNase and DNase-free tubes, frozen in liquid nitrogen, and then stored at − 80 °C for further analysis. This study was approved by the Ethics Committee of the Research Institute of Endocrine Sciences, Shahid Beheshti University of Medical Sciences (IR.SBMU.ENDOCRINE.REC.1395.216). Moreover, we conducted the current study in accordance with the Declaration of Helsinki and RIES institutional guidelines. Written informed consent was obtained from all participants.

### Genomic DNA extraction

Genomic DNA was isolated according to the manufacturer’s instructions from the fresh frozen thyroid specimens using the FavorPrep Tissue Genomic DNA Extraction Mini Kit, USA. In order to determine DNA quality, ND-1000 spectrophotometer (Thermo Scientific, USA) absorbance ratios between 260 and 230 nm were determined, which were within acceptable parameters.

### Design of primers

**Fig. 1:**
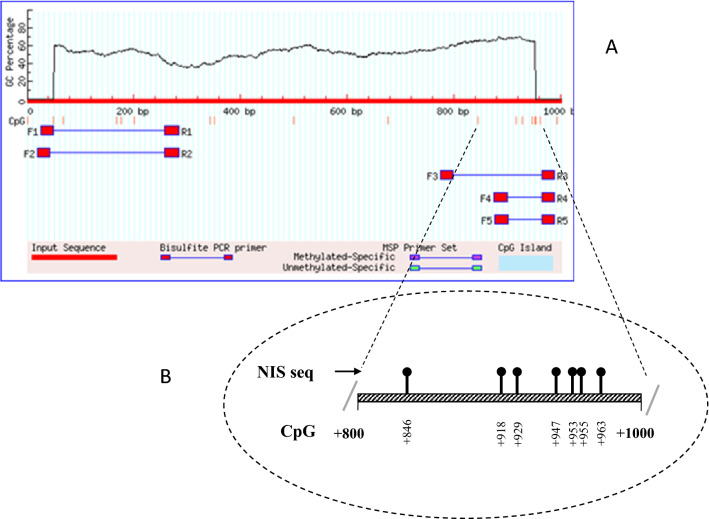
Methylation status in NIS promoter. **a** The probable promoter sequence checked for the presence of the most frequent CpG islands for the NIS gene and suggested primers from the MethPrimer website. **b** Structure of the human NIS promoter gene and the selected seven CpG positions

Met-primer online Software was used to design primers for hot-start PCR. These primers were designed for the promoter region of *NIS* gene (Table 1). In summary, 1000 nucleotides upstream of the start codon were selected from genome databases. Islands in the promoter were detected according to the specific inclusion criteria, including island size > 100, GC percent > 50.0, and obs/exp > 0.6, CpG. Finally, we found seven CpG sites for *NIS* promoter (CpG1-7 including +846, +918, +929, +947, +953, +955, and +963, respectively) and investigated methylation status in these sites (Fig. 1). This was the only upstream region in the characterized promoter that was CpG-rich. In order to evaluate the expression of *NIS* gene, primers for qRT-PCR were designed using GeneRunner software (version 4.0) and checked in NCBI Primer Blast.

**Table 1:** The information of methylation and qRT-PCR primers

Genes	Accession number	Sequence 5′ → 3′	Start	Tm (°C)	Product size (bp)	No. CpG
Methylation primers						
NIS	NG_012930.1	F:GTGATTAGGGGATTATAGTGTATGG	− 775	57.13	210	7
		R:TAAATTACAAATTTATTAAACTCCC	− 988	52.69		
qRT-PCR primers						
NIS	NM_000453.3	F:CCCAGACCAGTACATGCCTC	–	59.82	86	–
		R:TGTAAGCACAGGCCAGGAA		58.85		
Β-actin	NM_001101.5	F:GATCAAGATCATTGCTCCTCCT	–	57.65	108	–
		R:TACTCCTGCTTGCTGATCCA		58.43		

*Tm* temperature

### Bisulfite modification and quantitative methylation detection (bisulfite sequencing PCR (BSP))

According to the manufacturer's protocol, bisulfite conversion was performed using the EZ DNA Methylation-gold kit (Zymo Research, USA). The sodium bisulfite-treated genomic DNA was amplified by hot-start PCR with the TEMPase HotStar Master Mix kit (Amplicon, UK). The PCR conditions were as follows: initial step at 95 °C for 3 min, followed by 35 cycles at 95 °C for 30 s, annealing at 55–60 °C for 30 s, elongation at 72 °C for 30 s, and the final elongation at 72 °C for 3 min. Direct DNA sequencing was used to determine the methylation pattern. The primers and purified PCR products were transferred to Kowsar Biotech Company (KBC, Iran, Tehran), using the power read DNA sequencing service. Sequencing results were received in chromatograph, FASTA, SEQ, and pdf formats and were analyzed using Chromas version 2.6.5. The methylation percentage at each position and for each sample was calculated by mC/(mC+C) formula for all examined CpGs; following that, the methylation percentage was also calculated at each CpG site.

### Extraction of RNA and synthesis of cDNA

After histological control, total RNA was extracted from fresh snap-frozen tissue samples using the TRIzol reagent (Ambion, USA), according to the manufacturer’s instructions. Following that, we used the Nanodrop spectrophotometer (ND-1000, Thermo Scientific, USA) to measure the 260/280 and 260/230 nm absorption ratios to assess the RNA quality, and the ratios were within an acceptable range. Finally, total RNA was reverse transcribed with the cDNA synthesis kit (Bio Fact, USA) according to the manufacturer’s protocol in a SENSOQUEST, Germany thermocycler.

### qRT‐PCR

To evaluate the *NIS* expression, quantitative reverse transcriptase real-time PCR (qRT-PCR) was performed using the Rotor-Gene 6000 (Corbett Research, Sydney, Australia). All experiments were performed duplicate for each sample in a total volume of 25 µl using the SYBR Green master mix (Bio Fact, Korea).

### Statistical analysis

Graph Pad Prism 8.0.1 and SPSS version 20 was used for statistical analyses. Kolmogorov–Smirnov-Test was employed to test the normal distribution of the data. Non-normally distributed variables were analyzed with the Wilcoxon rank test to assess paired comparisons and Mann–Whitney U test for unpaired comparisons. Methylation results are presented with mean and standard error measurements of the mean. The mRNA expression results are expressed with Median and interquartile (25th, 75th percentiles). For all comparisons, P-values < 0.05 were considered statistically significant. Relative quantitation of mRNA levels of *NIS* was performed by the comparative Ct method using the 2^−ΔΔCT^ method [21]. The relationship between the expression and total methylation of *NIS* in tumoral tissues of PTC and FTC patients was examined by calculating Pearson correlation coefficients.

## Systematic literature search

The current systematic review was designed according to the latest version of the PRISMA checklist for systematic review [22]. A literature search by using the terms including “Thyroid Neoplasm” OR "Thyroid Carcinoma" OR "Thyroid Cancer" AND "DNA Methylations” OR “hypermethylation" OR "CpG Island" AND “sodium iodide symporter” OR "*SLC5A5*" was conducted in PubMed/MEDLINE, Web of Science, and Scopus databases. The search was performed without restricting publication date. Only articles in English were evaluated. The search results were managed by EndNote version X7. Titles and abstracts were reviewed, and relevant studies were selected. Inclusion criteria were as follows: The study indicated methylation of the *NIS* gene promoter in differentiated thyroid cancer (PTC and FTC). Then the full-text versions of selected articles were retrieved. The unrelated studies, reviews, meta-analysis, whole-genome, and wide genome studies were excluded. However, the references of related reviews were checked to identify additional papers. The following characteristics were collected from included studies: authors, year of publication, sample size groups, methodology, methylation status, and potential clinical values.

## Results

### Demographic and clinicopathological characteristics

The mean ± SD of age in PTC, FTC, and MNG groups were 37.89 ± 12.17, 55 ± 18.15, and 48.67 ± 14.75 years, respectively (P = 0.001). The Mean ± SD of tumor sizes were 1.62 ± 0.96 and 3.26 ± 1.68 cm in PTC and FTC groups, respectively (P = 0.002). Blood vascular invasion and TNM stages were significantly different between PTC and FTC groups (P = 0.007 and P = 0.001, respectively). However, there was no significant difference in the extracapsular invasion, extrathyroidal extension, and lymph node metastasis between PTC and FTC patients (P = 0.057, P = 0.068, and P = 0.407, respectively). The demographic and clinicopathological characteristics of patients are shown in Table 2.

**Table 2:** Demographic and clinicopathological characteristics of patients

Parameters	PTC	FTC	MNG	Total
Patients number	28	9	27	64
Gender				
Male	2 (7.1)	4 (50)	8 (29.6)	14 (22.2)
Female	26 (92.9)	4 (50)	19 (70.4)	49 (77.8)
Age (years)				
< 45	21 (75)	3 (37.5)	8 (29.6)	32 (50.8)
≥ 45	7 (25)	5 (62.5)	19 (70.4)	31 (49.2)
BRAF V600E mutation				
Positive	12 (46.2)	2 (22.2)	–	–
Negative	14 (53.8)	7 (77.8)	–	–
Tumor size (cm)				
< 2	13 (46.4)	2 (25)	–	–
≥ 2	15 (53.6)	6 (75)	–	–
Extrathyroidal extension				
Positive	4 (14.8)	3 (42.9)	–	–
Negative	23 (85.2)	4 (57.1)	–	–
Extracapsular invasion				
Positive	4 (14.8)	3 (42.9)	–	–
Negative	23 (85.2)	4 (57.1)	–	–
Lymph node metastasis				
Positive	8 (29.6)	1 (14.3)	–	–
Negative	19 (70.4)	6 (85.7)	–	–
Blood vascular invasion				
Positive	4 (14.8)	2 (71.4)		
Negative	23 (85.2)	5 (28.6)		
TNM stage (AJCC)				
I and II	23 (82.1)	4 (57.1)	–	–
III and IV	5 (17.9)	3 (42.9)	–	–

*PTC* papillary thyroid cancer, *FTC* follicular thyroid cancer, *MNG* multinodular goiter, *AJCC* American Joint Committee on Cancer

### Analysis of NIS mRNA expression and its promoter methylation in PTC patients

**Fig. 2:**
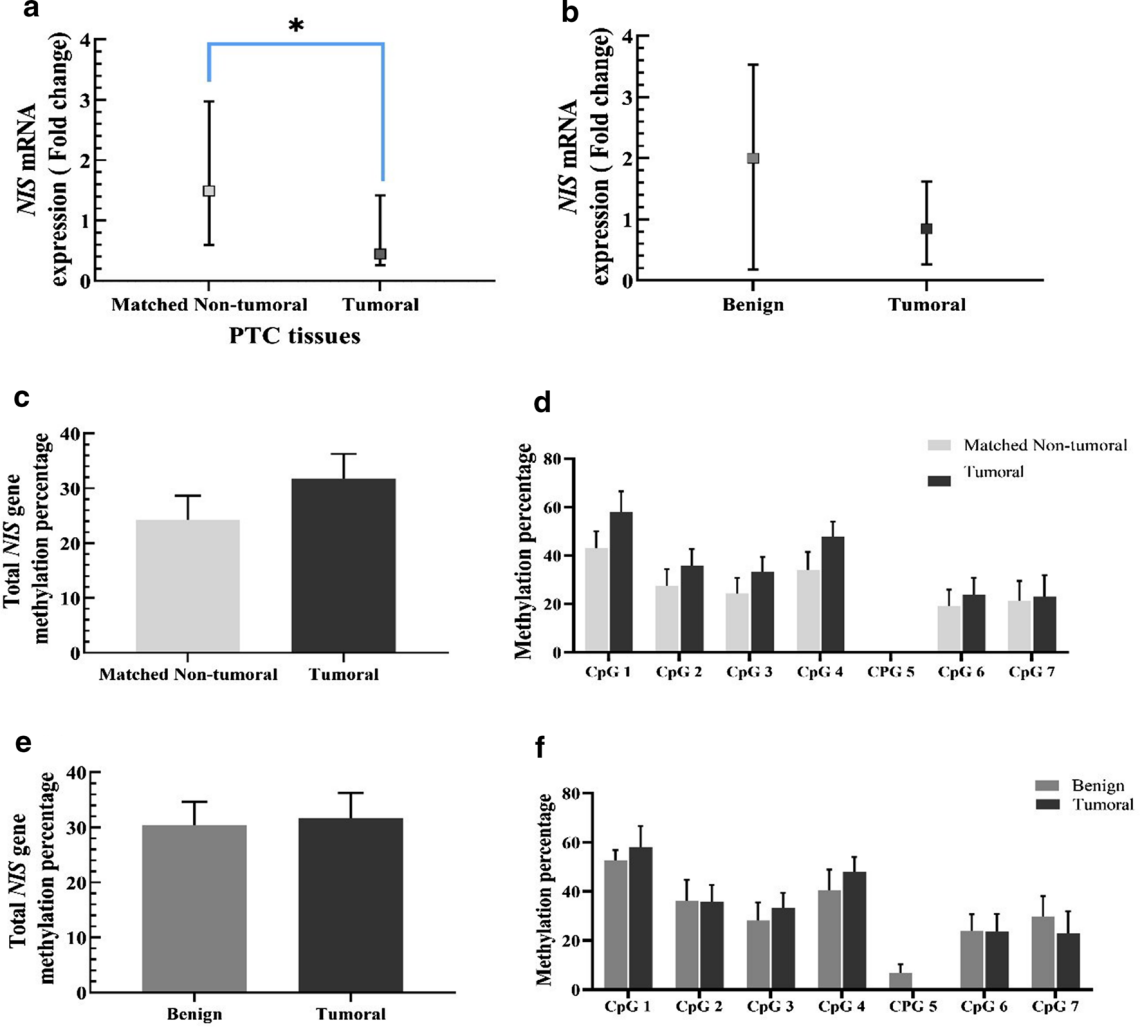
**a** *NIS* levels in PTC tissues compared to matched non-tumoral tissues. **b** *NIS* levels in PTC tissues compared to MNG tissues. Data are presented as median (CI 95%). **c** Total methylation of *NIS* promoter in PTC tissues compared to the methylation degree in matched non-tumoral tissues. **d** the methylation percentage of seven CpGs sites of *NIS* promoter in PTC tumoral compared to matched non-tumoral tissues. **e** total methylation percentage of *NIS* promoter in PTC tumoral compared to the MNG tissues. **f** the methylation percentage of seven CpGs sites of *NIS* promoter in PTC tumoral compared to MNG tissues. Data are presented as Mean ± 1SEM

The results showed that the *NIS* mRNA level was significantly reduced in PTC tumoral tissues compared to their matched non-tumoral tissues (0.45 *vs* 1.49, respectively, P = 0.04, Fig. 2a). There were no significant changes in the expression of *NIS* mRNA levels between PTC patients and MNG participants (0.84 *vs* 2.00, P = 0.76, Fig. 2b). The findings demonstrated no significant differences between total methylation status in PTC and the matched non-tumoral tissues (31.71 ± 4.54 vs 24.25 ± 4.39, P = 0.10, Fig. 2c). We also performed the analysis in each CpG site, and the differences were not significant in none of the CpG sites (Fig. 2d). In addition, no significant differences were observed in total methylation status (31.69 ± 4.56 vs 30.41 ± 4.24, P = 0.61, Fig. 2e), and none of the CpG sites between PTC and MNG tissues (Fig. 2f).

### Analysis of NIS mRNA expression and its promoter methylation in FTC patients

**Fig. 3:**
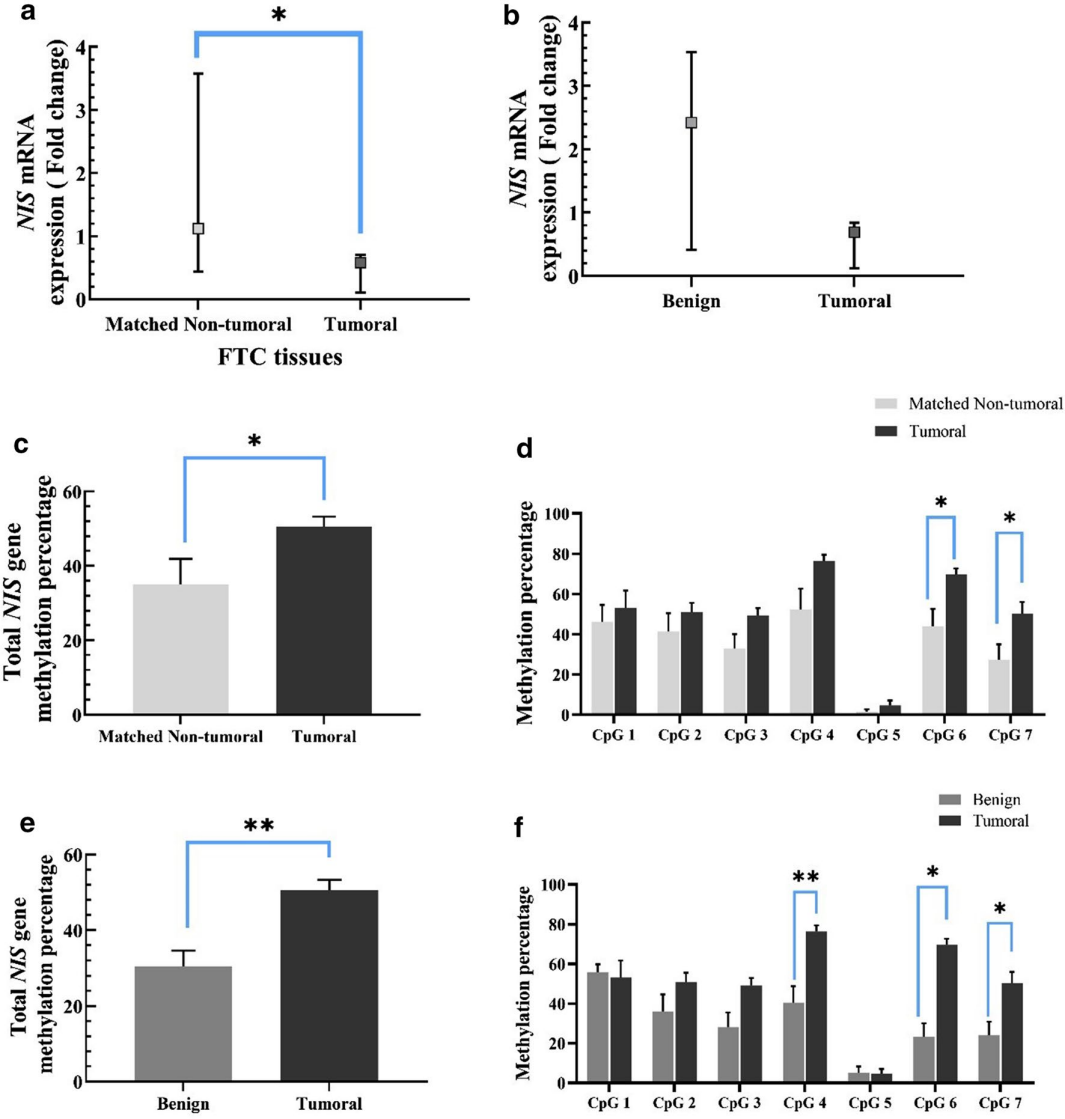
**a** *NIS* levels in FTC tissues compared to matched non-tumoral tissues. **b** *NIS* levels in FTC tissues compared to MNG tissues. Data are presented as median (CI 95%). **c** Total methylation of *NIS* promoter in FTC tissues compared to the methylation degree in matched non-tumoral tissues. **d** The methylation levels in FTC tumoral and the matched non-tumoral of 6th (+ 955) CpG site and of 7th (+ 963) CpG site. **e** Total methylation percentage of *NIS* promoter in FTC tumoral compared to the MNG tissues. **f** The methylation degrees in the 4th (+ 947), 6th (+ 955), and 7th (+ 963) CpG sites in the forward strand of *NIS* promoter between FTC tumoral and MNG tissues. Data are presented as Mean ± 1SEM

The *NIS* expression level was significantly reduced in FTC tumoral tissues compared to the matched non-tumoral tissues (0.53 *vs* 1.11, P = 0.03, Fig. 3a). There were marginally differences in the expression of *NIS* mRNA levels between FTC patients and MNG participants (0.69 *vs* 2.42, P = 0.07, Fig. 3b). Total methylation degree was substantially increased in FTC compared to the matched non-tumoral tissues (50.6 ± 2.67 *vs* 35.06 ± 6.83, P = 0.03, Fig. 3c). The methylation levels in FTC tumoral and the matched non-tumoral were found to be 69.63 ± 3.03 *vs* 43.92 ± 8.63 for the 6th (− 955) CpG site (P = 0.03) and 50.33 ± 5.65 *vs* 27.21 ± 7.68 for 7th (− 963) CpG site (P = 0.02) (Fig. 3d). However, total methylation levels of FTC tumoral tissues were considerably higher than MNG tissues (50.6 ± 2.68 *vs* 30.41 ± 4.24, P = 0.005, Fig. 3e). Further, significant differences were observed in the methylation levels in the 4th(− 947), 6th(− 955), and 7th(− 963) CpGs sites in the forward strand of *NIS* promoter between FTC tumoral and MNG tissues (76.34 ± 3.12 *vs* 40.43 ± 8.42, P = 0.004, 69.63 ± 3.03 *vs* 23.29 ± 6.84, P = 0.001 and 50.33 ± 5.65 *vs* 24 ± 6.89, P = 0.030, respectively, Fig. 3f).

### Correlation between mRNA expression and total methylation

**Fig. 4:**
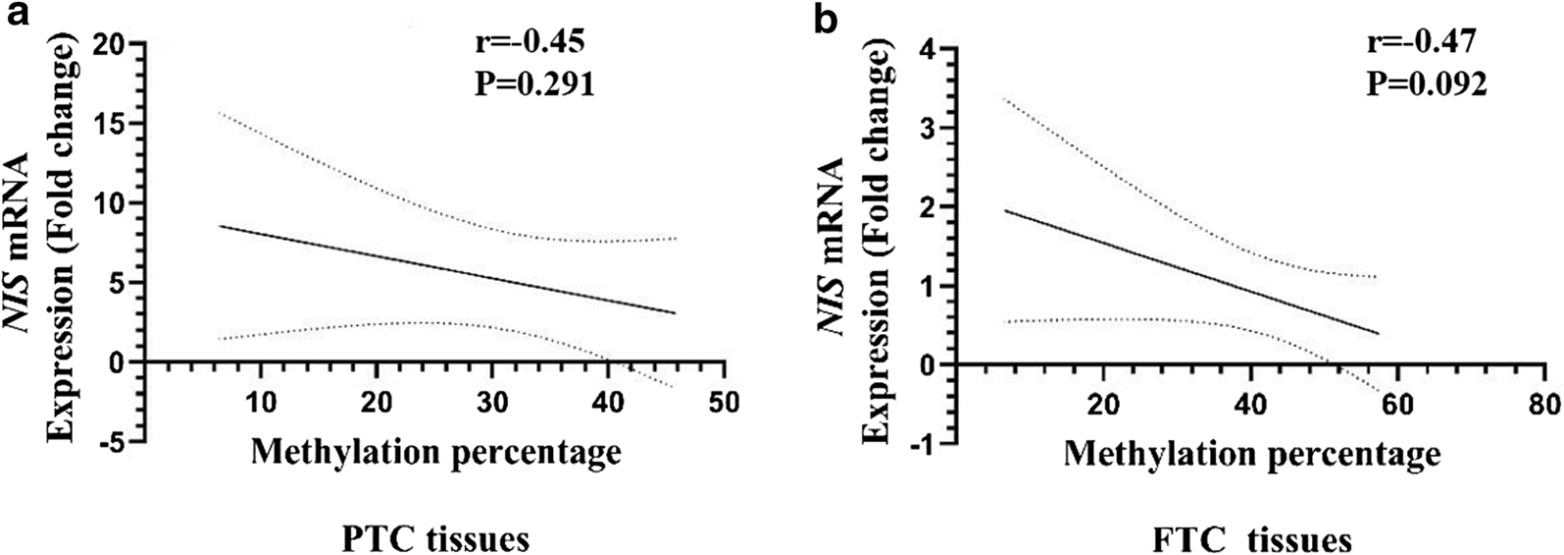
The correlation between the expression and total methylation of tumoral tissues of **A** PTC and **B** FTC patients for *NIS* genes

There was no significant correlation between *NIS* mRNA expression and methylation status in PTC (R^2^ = 0.23, P = 0.229), while a marginal significant correlation was observed in FTC (R^2^ = 0.27, P = 0.092) (Fig. 4).

### Systematic literature results

**Fig. 5:**
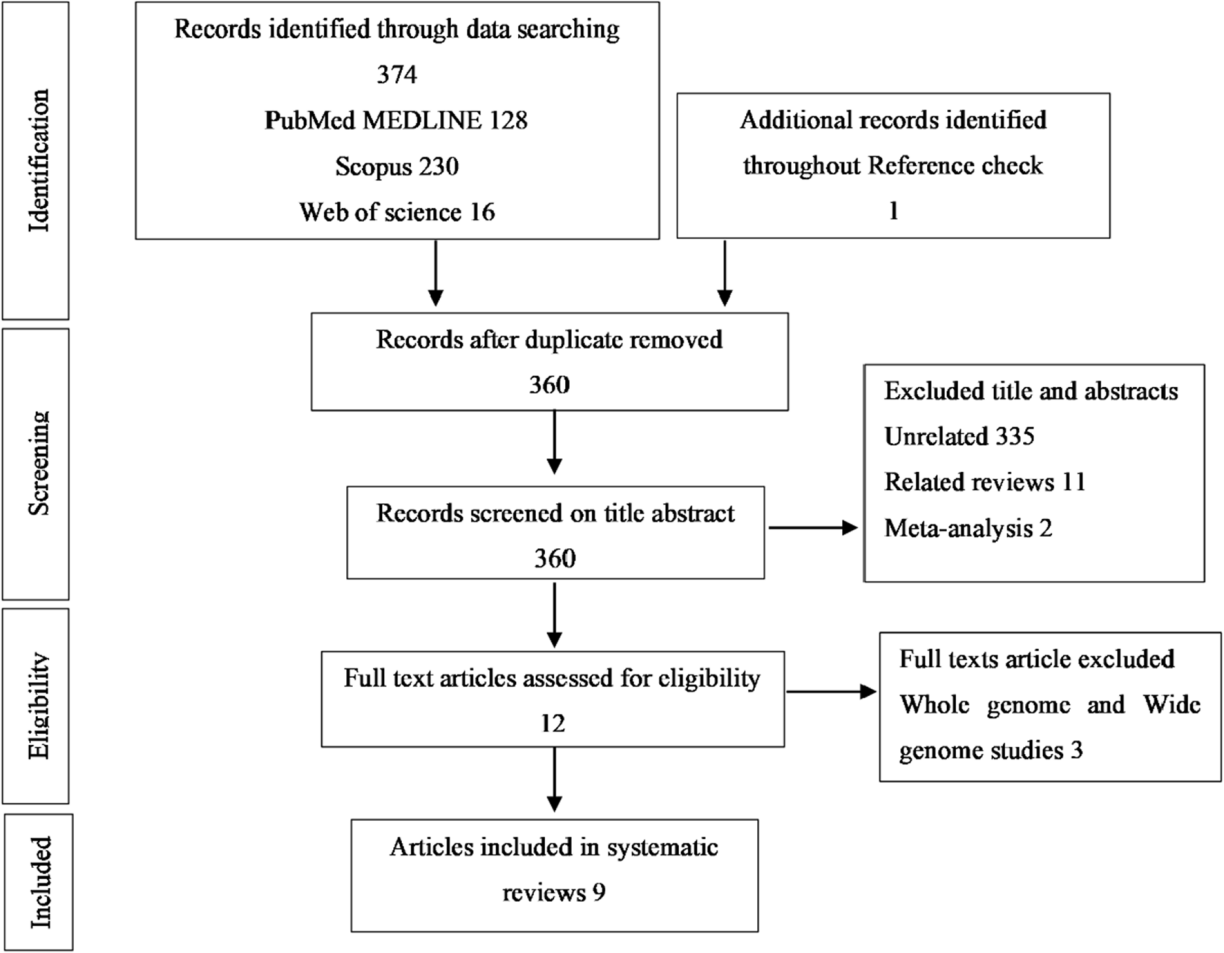
PRISMA flowchart detailing the selection of publications for this systematic review

The literature search revealed 374 papers. After removing duplicates, 360 have remained. 348 studies were excluded based on inclusion criteria in the screening process, and the remaining related articles were selected to review the full-text paper. Finally, 9 studies met the eligibility criteria and were chosen to evaluate promoter methylation of differentiated thyroid cancer for *NIS* gene (Fig. 5).

## Discussion

Thyroid cancers are the most common endocrine malignancies, which comprise several subtypes; among them, PTC and FTC are referred to WDTCs, accounting for more than 90% of all thyroid malignancies [23]. It is well accepted that increased methylation may induce dedifferentiation and decrease the expression of thyroid tissue-specific genes. DNA methylations are heritable and reversible; it is speculated that target therapy may alter methylation status of these genes to achieve the goal of tumor treatment. In this regard, the present study was designed to determine the methylation status of *NIS* promotor in thyroid nodules.

*NIS* is a transmembrane glycoprotein mainly regulated by the *TSH* that mediates the active transport of iodide from the bloodstream into the follicular thyroid cells [24]. The CpG islands in the *NIS* gene promoter have been suggested as a methylation target in reducing gene expression [25]. This study showed that although the expression of *NIS* was significantly lower in PTC tumoral tissues compared to matched non-tumoral ones, the methylation status was not changed significantly in any of the CpG sites. Moreover, no significant differences were observed between the mRNA levels and methylation patterns in PTC tumoral lesions compared to the benign MNG group. Further, our results indicated that the mRNA level of *NIS* was significantly reduced in FTC tumoral tissues compared to both matched non-tumoral and benign MNG lesions. Besides, FTC tumoral tissues displayed significantly higher levels of *NIS* promoter methylation compared to non-tumoral and benign MNG tissues. These findings were consistent with the hyper-methylation of the 4th, 6th, and 7th CpG positions in the forward strand of the *NIS* promoter.

Our systematic research achieved nine studies that explored *NIS* gene promoter methylation status in normal and tumoral thyroid tissues and benign lesions (Table 3). Some studies emphasize an increase in *NIS* promoter methylation in malignant tissues, while others showed controversial results. In 1999, Venkataraman et al*.* [14] assessed the methylation of the *NIS* promoter in 100 bp upstream and downstream, extending to the first intron, of CpG-rich dinucleotides (named P, L, C regions) and its expression in PTC and FTC tumor tissues. Our results are in line with their findings, which showed that methylation in three regions was not associated with NIS loss in PTC tumors. A CpG rich segment of the coding region (region C) displayed heterogeneous methylation among PTC subjects without any correlation to *hNIS* mRNA expression. Similarly, FTC cases expressed NIS mRNA and had different methylation patterns between regions. Nevertheless, 5-azacytidine or sodium butyrate treatment of seven human thyroid carcinoma cell lines was found to restore NIS mRNA expression in four cell lines and iodide transport in two cell lines. In a study by Neuman et al*.* [26], a 42.8% (6/14) occurrence of *NIS* methylation was found in cold thyroid nodules (CTNs) without determining the type of thyroid cancer relative to non-methylation in their non-tumoral surrounding tissues. Consistent with our findings, they detected a significantly reduced *NIS* expression ranging from 3 to 68% compared to the corresponding surrounding tissues (ST) in 86% of the CTNs. Moreover, they did not observe significant correlation between *NIS* mRNA expression and the degree of methylation. In another study conducted by Smith et al. [27], increased methylation of *NIS* in 22% (7/13) of patients with PTC and 0% of the control group, including follicular adenomas (FA), goiters, and normal cases were observed. However, the author pointed out that the limited number of the study groups led to not concluding predictive value from the comparison. In our findings, methylation of the *NIS* gene was increased in all regions except the 5thCpG site in PTC tumoral tissues compared to adjacent normal tissues, while this elevation was not statistically significant. A pilot study by Stephen et al*.* in 2011 [28] showed *NIS* promoter was methylated in (7/13) of DTCs (without specifying PTC or FTC separately), (1/3) of hyperthyroid, and (1/5) of normal cases and suggested that aberrant methylation of *NIS* may be an early change in thyroid tumorigenesis regardless of cell type. By applying semi-quantitative (SQ) MSP, Galaro et al*.* [12] investigated the methylation levels of the *NIS* CpG island between nucleotide − 466 and + 246 from the ATG in fragmented regions. They showed that the 5′ upstream region of the CpG island had the highest methylation levels, decreasing toward the 3’ region (P1 < P2 < L < C, p < 0.05). Besides, similar degree of methylation was reported in PTC tumoral tissues (benign = 2.19 ± 0.50 AU, malignant = 1.92 ± 0.59 AU) and non-tumoral tissues (benign = 2.23 ± 0.58 AU, malignant = 2.20 ± 0.82 AU) and suggested that *NIS* promoter methylation is a common occurrence in benign and malignant tumors as well as in the surrounding tissues. In agreement with our result, no quantitative correlation was detected between methylation levels and mRNA expression in any of the groups. Later, Galaro et al*.* in 2014 [16] appraised two CpG islands; the first had been described by Venkataraman et al*.* [14], which was referred to as CpG-island1, and the second, the new NIS CpG-island2 with 14 CpG sites, comprised a sequence of 256 bp and located at − 2152/− 1887 relative to the ATG site. They found a novel distal enhancer regulating the mRNA expression of *NIS* through DNA methylation, then by using QBS-sequencing, indicating that the methylation degree of 66.1% in tumoral tissues (benign, 64.3%, and malignant, 69.6%), compared to 23.2% of none tumoral (NT) tissues (benign, 20.5%; malignant, 25.1%). In contrast to our result, a significant inverse correlation between NIS mRNA expression and the degree of CpG-island2 methylation in NT and T samples was observed. At the same year, Choi et al. (2014) [29], found more frequent *NIS* methylation, in six specific CpG sites in the promoter region (− 191 to + 73), in cases of *BRAF V600E* carrier PTC samples than surrounding normal (SN) tissues (normal 29% *vs.* cancer 58%). In particular, − 24 and − 26 CpG sites were highly methylated in *BRAF V600E* PTCs.

**Table 3:** Characteristic collected from included studies

Studies	Method	Studied groups	Methylation status			Potential Clinical Value
			NT	BTLs	DTCs	
Venkataraman et al*.* (1999)	MSP	NT (5), PTC (16), FTC (2)	2/5	–	8/18	Chemical demethylation therapy
Neuman et al*.* (2004)	MSP	ST (14), CTN (14)	0/14	–	6/14	Could be a regulatory mechanism of NIS transcription
Smith et al*.* (2007)	MSP	Controls (FA (10), goiters (15), normal thyroids (2)), PTC (32)	0	0	7/32	Marker of virulence
Stephen et al*.* (2011)	MSP	NT (5), hyperthyroid (3), PTC (11), FTC (2)	1/5	1/3	7/13	Early event
Galrão et al*.* (2013)	SQ-MSP	BTLs (10), PTC (18), FTC (2)	–	2.23	1.92	Very frequent event
Galrão et al*.* (2014)	BSP	BTLs (10), PTC (18), FTC (2)	–	23.2%	66.1%	Early event
Choi et al*.* (2014)	BSP and MSP	NT (24), PTC (24)	29%	–	58%	Related to BRAF (V600E) mutation
Stephen et al*.* (2015)	QMSP	ANT (24), FTC-Hurthle (26), FTC-Classic (27)	> 0.0	–	> 0.0	Early changes in thyroid tumorigenesis regardless of cell type
Stephen et al*.* (2018)	QMSP	NT (71), FA (83), PTC (85), FTC (90)	0.001(0.002)	0.014 (0.041)	PTC (0.009(0.027)) FTC (0.02(0.07))	–

Methylation status of *NIS* gene promoter: MSP, SQ-MSP, BSP, and QMSP results were expressed as methylated/total samples, arbitrary unit (AU), percentage, and mean (SD), respectively

*MSP* methylation-specific PCR, *SQ-MSP* semi-quantitative MSP, *QMSP* quantitative MSP, *BSP* Bisulfite-sequencing PCR, *ST* surrounding tissue, *CTN* cold thyroid nodules, *NT* normal thyroid, *FA* follicular adenoma, *BTLs* benign thyroid lesions, *TC* thyroid cancer, *FTC* follicular thyroid cancer, *PTC* papillary thyroid cancer, *DTC* differentiated thyroid cancer, *ANT* adjacent normal tissue

According to the literature, methylation status of *NIS* gene in FTC tumoral tissues compared to matched non-tumoral and benign tissues is limited. In 2015, Stephen et al. [30] investigated the promoter methylation status of *NIS* gene related to thyroid cancers in a cohort of FTC compared to 26 Hurthle and 27 classic subtypes using the QMSP method. *NIS* gene was methylated (QMSP values > 0.0), but inversely to our findings, the methylation degrees were not statistically significant between different subtypes. They also reported frequent methylation of *NIS* in normal thyroid samples, hyperthyroid nodules, and thyroid cancers, suggesting this is a signal of early thyroid tumorigenesis, regardless of cell type. Eventually, in the following, Stephen et al. in 2018 [31] found significant differences in methylation levels of *NIS* promoter between FTC and normal thyroid groups. These findings imply that the *NIS* promoter hyper-methylation may result in a reduction of its expression in FTC. In contrast to Stephen's [30] findings, we detected a significant decrease in *NIS* expression and an increase in promoter methylation status in FTC samples compared to their matched non-tumorous and benign samples, with no association between mRNA and methylation levels.

There are conflicting reports about *NIS* methylation status, mRNA level, and the correlation between these two events in limited studies with restricted sample sizes. Several reasons may contribute to the inconsistent results, including the use of different methods, study groups, and population, as well as the different CpG island regions used in the studies. Nonetheless, the present findings in PTC subjects seem to be consistent with Smith et al. [27], Galaro et al. [12], and Stephen et al. [31] reports, which found no significant differential methylation of *NIS* promoter in PTCs tumors. Due to the methods used, some studies did not specify the area investigated and results were just reported as methylated and non-methylated, consequently comparing the results is quite challenging.

According to our study, promoter methylation of the *NIS* is not a common event in PTC tumors, their matched non-tumor, and MNG tissues; consequently, promoter methylation of *NIS* could not be a possible mechanism leading to reducing its expression. Maybe other regulatory mechanisms that affect this gene’s expression during neoplastic transformations are involved. However, there are many other CpG sites in the *NIS* gene promoter and the lack of methylation difference in this region cannot be generalized to the overall percentage of promoter methylation. In fact, the study of methylation of all CpG promoter sites of this gene will provide valuable information. As well as, based on the literature, methylation status is heterogeneous in various CpG regions of NIS gene promoter; whether it is the upstream or downstream of the start codon (ATG) of the gene, subsequently, further research is needed in this area.

There are no distinct patterns of methylation linked with transcriptional failure. This shows that thyroid nodule methylation is heterogeneous. The absence of a distinct methylation pattern also implies the importance of other epigenetic processes, which may alter the production of *NIS* mRNA levels. We underlined the need for additional research to understand the epigenetic mechanisms underlying the reduction of *NIS* mRNA expression, which could lead to developing novel treatments to enhance radioiodine uptake in thyroid carcinoma cells. These investigations can also contribute to a better comprehension of how epigenetic events influence gene expression. Additionally, it is anticipated that the design of targeted demethylation medicines, which may play a role in the redifferentiation of thyroid carcinomas, will lead to a novel cancer therapeutic strategy.

DNA methylation as an epigenetic modification has improved the understanding of thyroid carcinogenesis. Much effort has been made to investigate the potential of drugs that block this form of epigenetic alteration to stimulate the re-expression of silenced genes in diverse malignancies. Lately, thyroid cancer management has been approached with more personalized medicine to prevent the over-diagnosis and over-treatment of tumors [32]. In addition, hypermethylation of thyroid-specific genes such as *NIS* is associated with the failure of clinical radioiodine treatment in thyroid cancer, one of the most common methods for treating DTCs [14]. A decrease in *NIS* expression will lead to a decrease in response to the treatment. Therefore, not only the investigations of methylation patterns in the *NIS* gene can be effective in choosing the type of treatment, but also in the prognosis of patients, it is expected that individuals with higher levels of methylation will respond less to iodine therapy, which ultimately leads to the design the effective drugs by reducing the methylation of this gene. There is little evidence of the effectiveness of demethylating agents in thyroid cancer. In the most studies, these drugs have been tested on cancer cell lines and have yielded promising results, however, have not yet been translated into clinical practice. But it may be used as a solo agent or combined with other drugs for proper therapy [33].

The strengths of the present study include this being the first study that determined the promoter methylation status in the selected region of *NIS,* a functional gene*,* in an Iranian population diagnosed with thyroid nodules. In order to obtain reliable results, and to avoid false-positive results from non-quantitative methods such as MSP, we evaluated *NIS* promoter methylation with a quantitative methodology. However, there were several limitations to this cross-sectional study, including the small sample size of patients and the non-achievable clinicopathological information. Time and budget constraints limited us from examining more CpG sites of this gene. According to the limited number of studies, further investigations with larger sample size and other sites that may be concerned with epigenetic regulation of *NIS* expression are required to elucidate the role of these mechanisms in the progression of thyroid nodules, leading to finding novel diagnostic biomarkers and improving the effectiveness of therapies.

## Conclusion

The present study provides additional evidence concerning *NIS* promoter methylation in thyroid nodules. Although we did not find significant differences regarding *NIS* methylation in PTC patients, the *NIS* mRNA expression was substantially decreased in PTC cases. While the methylation of this gene was increased in tumoral tissues compared to matched non-tumoral tissues and MNG one. Intriguing results were found for FTC cases about the methylation pattern of the sodium-iodide symporter in CpGs islands. These differences in aberrantly methylated CpGs between PTC and FTC subjects reflect a potentially interesting findings and provide the following insights for future research. One hypothesis that warrants further review is that promoter regions are methylated more frequently in FTC than in PTC in our study. This hypothesis cannot be confirmed with our modest number of cases, but it does raise questions that can be investigated further. The investigations can also contribute to a better understanding of how epigenetic events influence gene expression. It is anticipated that the design of targeted demethylation medicines, which may play a role in the dedifferentiation of thyroid carcinomas, will lead to a novel cancer therapeutic strategy.

## Data Availability

The datasets used during the current study available from the corresponding author on reasonable request.

## Code availability

Non-applicable.
